# Genomics of Staphylococcus aureus and Staphylococcus epidermidis from Periprosthetic Joint Infections and Correlation to Clinical Outcome

**DOI:** 10.1128/spectrum.02181-21

**Published:** 2022-06-28

**Authors:** Margarita Trobos, Rininta Firdaus, Karin Svensson Malchau, Jonatan Tillander, Dimitrios Arnellos, Ola Rolfson, Peter Thomsen, Iñigo Lasa

**Affiliations:** a Department of Biomaterials, Institute of Clinical Sciences, Sahlgrenska Academy, University of Gothenburg, Gothenburg, Sweden; b Center for Antibiotic Resistance Research (CARe), University of Gothenburg, Gothenburg, Sweden; c Department of Orthopedics, Institute of Clinical Sciences, Sahlgrenska Academy, University of Gothenburg, Gothenburg, Sweden; d Department of Orthopaedics, Sahlgrenska University Hospital, Region of Västra Götaland, Gothenburg, Sweden; e Department of Infectious Diseases, Sahlgrenska University Hospital, Region of Västra Götaland, Gothenburg, Sweden; f Institute of Biomedicine, Department of Infectious Diseases, University of Gothenburg, Gothenburg, Sweden; g 1928 Diagnostics, Gothenburg, Sweden; h Laboratory of Microbial Pathogenesis, Navarrabiomed, Hospital Universitario de Navarra (HUN), Universidad Pública de Navarra (UPNA), IdiSNA, Pamplona, Spain; Riverside University Health System, Medical Center, University of California

**Keywords:** *Staphylococcus*, periprosthetic joint infection, biofilm, antibiotic resistance, virulence, patient outcome, whole-genome sequencing

## Abstract

The approach of sequencing or genotyping to characterize the pathogenic potential of staphylococci from orthopedic device-related infection (ODRI) has been applied in recent studies. These studies described the genomic carriage of virulence in clinical strains and compared it with those in commensal strains. Only a few studies have directly correlated genomic profiles to patient outcome and phenotypic virulence properties in periprosthetic joint infections (PJIs). We investigated the association between genomic variations and virulence-associated phenotypes (biofilm-forming ability and antimicrobial resistance) in 111 staphylococcal strains isolated from patients with PJI and the infection outcome (resolved/unresolved). The presence of a strong biofilm phenotype in Staphylococcus aureus and an antibiotic-resistant phenotype in Staphylococcus epidermidis were both associated with treatment failure of PJI. In S. epidermidis, multidrug resistance (MDR) and resistance to rifampicin were associated with unresolved infection. Sequence type 45 (ST45) and ST2 were particularly enriched in S. aureus and S. epidermidis, respectively. S. epidermidis ST2 caused the majority of relapses and was associated with MDR and strong biofilm production, whereas ST215 correlated with MDR and non/weak biofilm production. S. aureus *agr* II correlated with resolved infection, while S. epidermidis *agr* I was associated with strong biofilm production and *agr* III with non/weak production. Collectively, our results highlight the importance of careful genomic and phenotypic characterization to anticipate the probability of the strain causing treatment failure in PJI. Due to the high rate of resistant S. epidermidis strains identified, this study provides evidence that the current recommended treatment of rifampicin and a fluoroquinolone should not be administered without knowledge of the resistance pattern.

**IMPORTANCE** This study addresses the presence and frequency of particular genetic variants and virulence factors found in staphylococcal bacteria causing periprosthetic joint infection (PJI) of the hip and knee to ascertain their clinical relevance as predictors of treatment failure. We characterized the genetic virulence traits of a large collection of clinical staphylococci isolated from patients with PJI and evaluated their association with the patient’s infection outcome. The results showed that S. aureus strains that produced strong biofilms and S. epidermidis strains with resistance to several antibiotics associated significantly with unresolved infection. Some particular genetic variants associated with biofilm formation and multidrug resistance. These traits should be considered important risk factors for the diagnosis and treatment guidance in PJI.

## INTRODUCTION

Prosthetic joint infection (PJI) is the most devastating complication of total hip arthroplasty (THA) (1). The infection leads to prolonged immobility, higher health care and societal costs, and many therapeutic challenges, including psychological distress (2–4). The current treatment of hematogenous and early-onset PJI includes debridement, antibiotics, and implant retention (DAIR), with success rates between 37% and 87% (5, 6). Our previous study showed that strong biofilm production was associated with a 5-fold increased risk for developing recurrent infections. Considering that current therapies for the elimination of established biofilms are overall insufficient, the best infection control strategy for PJI is the prevention of bacterial adhesion to the surface of the implant, either by repelling bacterial cells from attaching (antifouling) or killing the attached bacteria in contact with the surface (bactericidal surfaces) (7). The effectiveness of either strategy is highly dependent on the capacity of the specific bacterial isolate to adhere to the surface and the microbial susceptibility profile. Thus, precise genomic and phenotypic characterization of strains producing PJI are important to identify associations between specific genomic traits and the probability of the strain to cause unresolved infections.

PJIs of the hip and knee are caused mainly by Staphylococcus aureus and Staphylococcus epidermidis (8, 9). Both species are commensals on human skin and mucous membranes and are able to adhere to and grow on prosthetic implants as biofilms (10, 11). In biofilms, bacteria grow embedded in extracellular polymeric substances (EPSs) made of polysaccharides, extracellular DNA, and proteins (12). The matrix helps to protect the bacteria from immunological (13) and pharmacological eradication (14–16). The development of biofilms includes four stages, as follows: attachment, accumulation, maturation, and dispersal. Each biofilm phase is mediated by species-specific genes. For example, S. aureus involves *atI*, *clfA*, *sarA*, and *spa* in attachment; *icaA*, *sarA*, and *spa* in accumulation; *arcA* in maturation; and *agr* and *hld* in dispersal; while S. epidermidis involves *atlE*, *embp*, *and sesC* in attachment; *bhp*, *embp*, *icaA*, and *sesC* in accumulation; *arcA* in maturation; and *agr* and *capB* in dispersal (11, 17).

The accessory gene regulator (Agr) is a quorum-sensing system in charge of regulating virulence traits, including biofilm development, depending on the density of the bacterial population (18–21). Mutation of *agr* in S. aureus and S. epidermidis results in the formation of thick biofilms. This phenotype has been associated with the downregulation of phenol soluble modulin (PSM) expression. PSMs disperse biofilms very likely due to their surfactant properties. Four *agr* groups of S. aureus (22) and three *agr* groups of S. epidermidis (23) have been reported, which are based on the amino acid sequence of the autoinducing peptide (AIP), encoded by the *agrD* gene.

Bacteria inside biofilms exhibit acquired and intrinsic antimicrobial resistance (AMR). Biofilms create an optimal environment for the horizontal transfer of AMR genes by plasmid exchange and mutation (24). The intrinsic AMR of biofilms is hypothesized to be due to reduced antibiotic diffusion through the EPS, reduced antibiotic uptake by the bacterial cell, and reduced metabolism (persister cells) (10, 25). A low growth rate makes sessile bacteria less vulnerable to cell wall-active antibiotics, and the biofilm matrix provides protection from immune cells (25). Methicillin-resistant S. aureus (MRSA) is commonly known for carrying *mecA*, which is spread through the SCCmec chromosome cassette, leading to resistance to non-β-lactam antibiotics (26). Penicillin resistance in S. aureus is caused by β-lactamase encoded by the plasmid-mediated *blaZ* gene (27).

The current understanding of the association between the molecular characteristics of S. aureus and S. epidermidis and the clinical outcome of PJIs is very limited. This study aimed to identify associations between genomic traits, biofilm formation capacity, and antibiotic susceptibility and the clinical outcome of PJI caused by S. aureus and S. epidermidis.

## RESULTS

### Patient outcome and clinical parameters.

The demographics of the study patient population are summarized in Table S1 in the supplemental material. A total of 111 strains of different staphylococcal species (45 S. aureus, 50 S. epidermidis, 11 Staphylococcus capitis, 2 Staphylococcus lugdunensis, 1 Staphylococcus simulans,1 Staphylococcus nepalensis, and 1 Staphylococcus pasteuri) belonging to 66 patients with PJI of the hip (*n* = 46) or knee (*n* = 21) (1 patient had a simultaneous bilateral hip and knee PJI) were included in the study. The majority of patients had mild (57.6%) to severe (34.8%) systemic comorbidities, according to the American Society of Anesthesiologists physical status class (ASA). Regarding the antimicrobial treatment regimens, all patients received intravenous (i.v.) therapy followed by oral therapy, and the mean duration of therapy (i.v. and oral) was 15.2 weeks. The most common type of i.v. treatment was vancomycin alone or in combination with another antimicrobial (68.1% of cases), and for oral treatment, rifampicin in combination with another antimicrobial agent was the most frequently used (60.6% of cases). Ten patients (15.2%) had a confirmed relapse, and 9 patients (13.6%) had reinfection with a different staphylococcal strain.

At the end of the study period (31 December 2018), approximately one-half of the patients (47%) had an unresolved outcome at the 3.5- to 5-year follow-up (Table 1). The unresolved outcome group commonly underwent 2 to 6 additional revision surgeries, whereas the patients with a resolved infection underwent none or one revision surgery (Table 1). As expected, patients with severe disease, as defined by ASA classes 3 and 4, were significantly more present in the unresolved infection group, whereas those with mild disease (ASA class 2) or healthy individuals (ASA class 1) were more present among the resolved infection group (Table 1). At the time of PJI diagnosis, patients with an unresolved infection had a significantly higher mean C-reactive protein (CRP) value (1.7-fold) than those whose infection was resolved (Table 1). While no differences were observed between patients infected with the different bacterial species (alone or in combination) and the infection outcome, patients infected with the more-virulent bacterial species S. aureus had a significant 3-fold increased CRP level than patients infected by the less-virulent S. epidermidis (Table 2).

**TABLE 1:** Association between prognostic factors and clinical outcome

Parameter^*a*^	Data by clinical outcome		*P* value^*b*^
	Unresolved	Resolved	
Total patients^*c*^ (*n* [%])	31 (47)	35 (53)	
Multiple revision surgery (*n* [%])			0.0003***^d^
No	7 (21.9)	25 (78.1)	
Yes	23 (71.9)	9 (28.1)	
Missing	1 (50)	1 (50)	
ASA class (*n* [%])			0.0235*^e^
Healthy	1 (25)	3 (75)	
Mild	14 (36.8)	24 (63.2)	
Severe	15 (65.2)	8 (34.8)	
Missing	1 (100)	0 (0)	
Inflammatory markers (mean ± SEM)			
ESR	58.6 ± 7.6	50.1 ± 6.2	0.3869^*f*^
WBC	9.1 ± 0.8	7.9 ± 0.4	0.2183^*f*^
CRP	130.1 ± 21.3	76.2 ± 15.3	0.0491*^f^
Bacterial species (*n* [%])			
S. aureus (*n *= 30)	12 (40)	18 (60)	0.5561^*d*^
S. epidermidis (*n *= 16)	8 (50)	8 (50)	0.7802^*d*^
S. aureus + S. epidermidis (*n *= 5)	3 (60)	2 (40)	0.5437^*d*^
Other CoNS (*n *= 8)	5 (62.5)	3 (37.5)	0.3478^*d*^
S. aureus + other CoNS (*n *= 1)	0 (0)	1 (100)	
S. epidermidis + other CoNS (*n *= 4)	2 (50)	2 (50)	0.9003^*d*^
S. aureus + S. epidermidis + other CoNS (*n *= 2)	1 (50)	1 (50)	0.9305^*d*^

a ASA, American Society of Anesthiologists physical status; ESR, erythrocyte sedimentation rate (mm/h); WBC, white blood cells (10^3^/μL); CRP, C- reactive protein (mg/L).

b Statistical significance is denoted as follows: *, *P* ≤ 0.05; **, *P* ≤ 0.01; ***, *P* ≤ 0.001.

c Each patient had ≥1 staphylococcal strain.

d Chi-square test.

e Cochran-Armitage trend test.

f Unpaired *t* test.

**TABLE 2:** Association between inflammatory markers and the bacterial species causing the infection

Parameter	Data for:		*P* value^*a*^
	S. aureus	S. epidermidis	
Total patients (*n*)	30	16	
Inflammatory markers (mean ± SEM)			
ESR	55.9 ± 30.3	52.3 ± 38.7	0.7982
WBC	8.9 ± 3.9	8.3 ± 2.7	0.6071
CRP	146.9 ± 107.9	49.3 ± 57.5	0.0016**^b^

a Unpaired *t* test.

b Statistically significant, *P* ≤ 0.01.

### Phylogenetic evaluation of the entire strain population based on cg-MLST.

**FIG 1:**
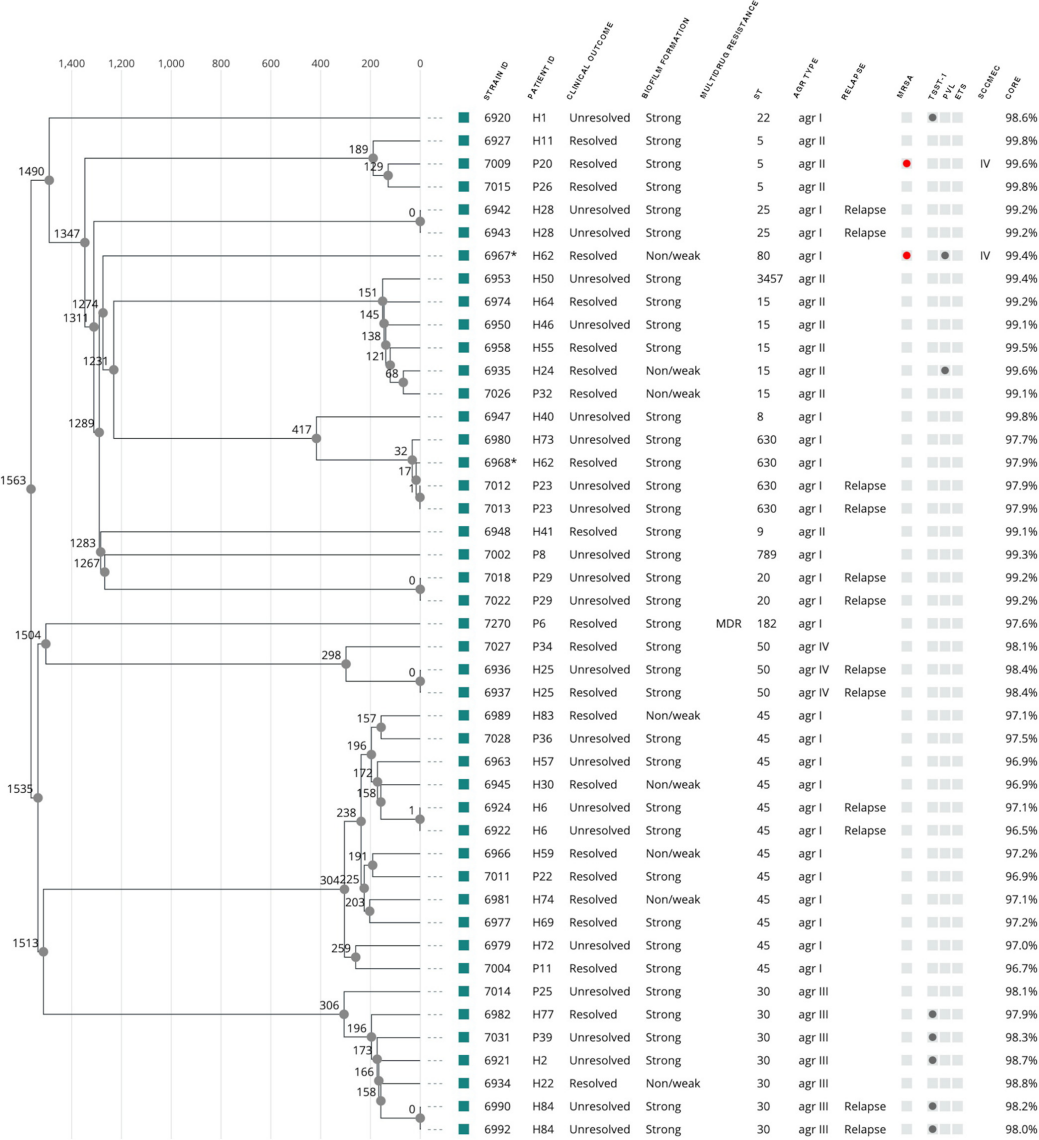
Phylogenetic relationships of S. aureus from PJI isolates based on core-genome multilocus sequence typing (cgMLST), phenotypic and genomic traits of strains, and patient infection outcome. Strain identifiers (IDs) that share same symbol (*) were coisolated the same date from the indicated patient ID. After the initial infection caused by strain 6936, patient H25 experienced a relapse with strain 6937 but no further infections were recorded during the rest of the study period. Therefore, patient H25 was classified as “unresolved infection” at isolation of 6936 and as “resolved infection” when 6937 was isolated. For the rest of patients experiencing a relapse, they were classified as unresolved infection since they experienced additional infections after the last isolated strain, as recorded in their medical journal. MDR, phenotypic multidrug resistance (≥3 antimicrobial agents); *agr*, accessory gene regulator; MRSA, methicillin-resistant Staphylococcus aureus; TSST-1, toxic shock syndrome toxin 1 (*tsst1*); PVL, Panton-Valentine leukocidins (*lukF-PVL*, *lukS-PVL*); ETS, exfoliative toxins (*etA*, *etB*); SCCMEC, Staphylococcal cassette chromosome *mec*; CORE, percentage of core genes found.

**FIG 2:**
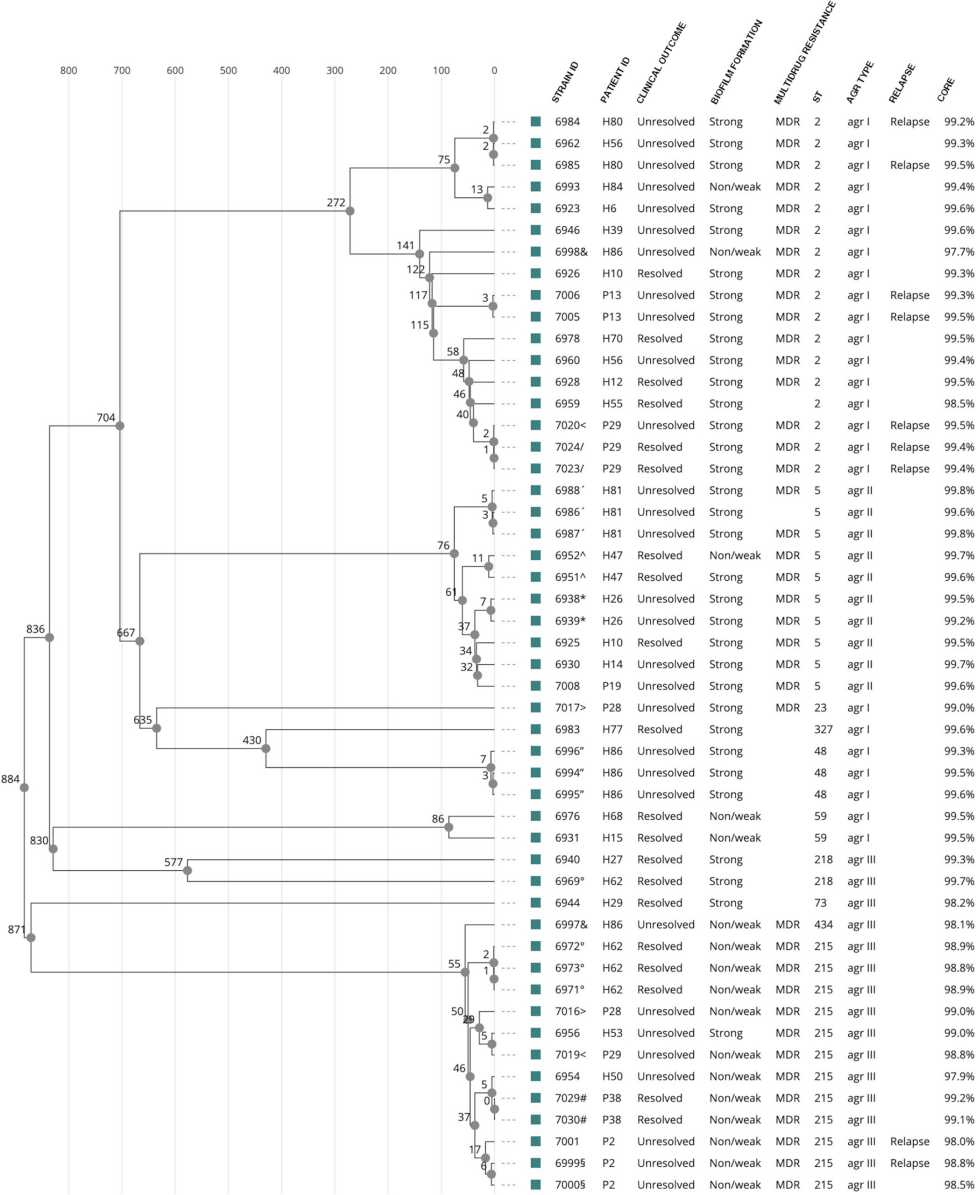
Phylogenetic relationships of S. epidermidis from PJI isolates based on core-genome multilocus sequence typing (cgMLST), phenotypic and genomic traits of strains, and patient infection outcome. Strain IDs that share same symbol were coisolated the same date from the indicated patient ID. After the initial infection caused by strain 7020, patient P29 experienced a relapse with strain 7023/7024, but no further infections were recorded during the rest of the study period. Therefore, patient P29 was classified as “unresolved infection” at isolation of 7020 and as “resolved infection” when 7023/7024 were isolated. For the rest of patients experiencing a relapse, they were classified as unresolved infection since they experienced additional infections after the last isolated strain, as recorded in their medical journal. MDR, phenotypic multidrug resistance (≥3 antimicrobial agents); *agr*, accessory gene regulator; CORE, percentage of core genes found.

We sequenced the genomes of 111 staphylococcal strains. Based on core genome multilocus sequence typing (cg-MLST) analysis of the sequenced genomes, the phylogenetic relationships between the 45 S. aureus strains and 50 S. epidermidis strains were visualized in a cgMLST-based phylogenetic tree showing the allele distances between the strains (Fig. 1, Fig. 2). The S. aureus strains clustered mainly by the sequence type (ST). The largest clusters in S. aureus were ST45 with 36.7% (12/45) of isolates and ST30 with 17.8% (8/45) of isolates (Fig. 1; Table 3). Due to the small sample size in each ST group, none of the ST types associated with the infection outcome, biofilm formation ability, or phenotypic MDR of the strain (Table 3).

**TABLE 3:** MLST of the S. aureus strains from PJI

ST^*a*^	CC	Total	Infection outcome			Biofilm formation			Multidrug resistance^*b*^^,^^*c*^	
			Unresolved^*c*^	Resolved^*c*^	*P* value	Non/weak^*c*^	Strong^*c*^	*P* value	Present	Not present
5	5	3 (6.7)	0 (0)	3 (100)		0 (0)	3 (100)		0 (0)	3 (100)
8	8	1 (2.2)	1 (100)	0 (0)		0 (0)	1 (100)		0 (0)	1 (100)
9	1	1 (2.2)	0 (0)	1 (100)		0 (0)	1 (100)		0 (0)	1 (100)
15	15	5 (11.1)	1 (20)	4 (80)	0.1399	2 (40)	3 (60)	0.1680	0 (0)	5 (100)
20		2 (4.4)	2 (100)	0 (0)		0 (0)	2 (100)		0 (0)	2 (100)
22	22	1 (2.2)	1 (100)	0 (0)		0 (0)	1 (100)		0 (0)	1 (100)
25		2 (4.4)	2 (100)	0 (0)		0 (0)	2 (100)		0 (0)	1 (50)
30	30	8 (17.8)	5 (71.4)	2 (28.6)	0.2163	1 (14.3)	6 (85.7)	0.7926	0 (0)	7 (100)
45	45	12 (36.7)	5 (41.7)	7 (58.3)	0.4447	4 (33.3)	8 (66.7)	0.0998	0 (0)	12 (100)
50		3 (6.7)	1 (33.3)	2 (66.7)	0.5237	0 (0)	3 (100)		0 (0)	3 (100)
80		1 (2.2)	0 (0)	1 (100)		1 (100)	0 (0)		0 (0)	1 (100)
182		1 (22)	0 (0)	1 (100)		0 (0)	1 (100)		1 (100)	0 (0)
630	8	4 (8.9)	3 (75)	1 (25)	0.3167	0 (0)	4 (100)		0 (0)	4 (100)
789	8	1 (2.2)	1 (100)	0 (0)		0 (0)	1 (100)		0 (0)	1 (100)
3457	15	1 (2.2)	1 (100)	0 (0)		0 (0)	1 (100)		0 (0)	1 (100)
Total		45 (100)	23 (51.1)	22 (48.9)		8 (17.8)	37 (82.2)		1 (2.2)	44 (97.8)

a ST, sequence type; CC, clonal complex; Total, total no. (%) of strains.

b Phenotypic multidrug resistance was considered when a strain was resistant to ≥3 antimicrobial agents.

c *n* (%).

The S. epidermidis strains also grouped by ST type showing three main clusters, with the largest being ST2, followed by ST215 and ST5 (Fig. 2). The ST2 S. epidermidis belonged to *agr* type I, ST215 was *agr* type III, and ST5 was *agr* type II (Fig. 2). Although no statistical significance was observed between S. epidermidis ST type and patient outcome (Table 4), ST2 caused the majority of relapses (Fig. 2).

**TABLE 4:** MLST of S. epidermidis strains from PJI^*a*^

ST	Total^*d*^	Infection outcome			Biofilm formation			Multidrug resistance^*b*^		
		Unresolved^*c*^	Resolved^*c*^	*P* value	Non/weak^*c*^	Strong^*c*^	*P* value	Present^*c*^	Not present^*c*^	*P* value
2	17 (33.3)	11 (64.7)	6 (35.3)	0.6259	2 (11.8)	15 (88.2)	0.0172**	16 (94.1)	1 (5.9)	0.0483*
5	10 (19.6)	7 (70.0)	3 (30.0)	0.4705	1 (10)	9 (90)	0.0733	9 (90)	1 (10)	0.3057
23	1 (2.0)	1 (100)	0 (0)		0 (0)	1 (100)		1 (100)	0 (0)	
48	3 (5.9)	3 (100)	0 (0)		0 (0)	3 (100)		0 (0)	3 (100)	
59	2 (3.9)	0 (0)	2 (100)		2 (100)	0 (0)		0 (0)	2 (100)	
73	1 (2.0)	0 (0)	1 (100)		0 (0)	1 (100)		0 (0)	1 (100)	
215	12 (23.5)	7 (58.3)	5 (41.7)	0.8925	11 (91.7)	1 (8.3)	<0.0001****	12 (100)	0 (0)	
218	3 (5.9)	0 (0)	2 (100)		0 (0)	2 (100)		0 (0)	2 (100)	
327	1 (2.0)	0 (0)	1 (100)		0 (0)	1 (100)		0 (0)	1 (100)	
434	1 (2.0)	1 (100)	0 (0)		1 (100)	0 (0)		1 (100)	0 (0)	
Total	50 (100)	30 (60)	20 (40)		17 (34)	33 (66)		39 (78)	11 (22)	

a *P* values were statistically significant at *, *P* ≤ 0.05; **, *P* ≤ 0.01; and ****, *P* ≤ 0.0001 by Chi-square test.

b Phenotypic multidrug resistance was considered when a strain was resistant to ≥3 antimicrobial agents.

c *n* (%).

d Total, total no. (%) of strains.

Regarding the relationship between *agr* typing and pathogenesis, S. aureus *agr* II strains were significantly associated with a resolved outcome (Table 5).

**TABLE 5:** Association between *agr* types, clinical outcome, biofilm formation, and multidrug resistance in S. aureus and S. epidermidis isolates^*a*^

Bacteria	*agr* type	No of isolates *n* (%)		*P* value	No of isolates *n* (%)		*P* value	Multidrug resistance^*b*^ *n* (%)		*P* value
		Unresolved	Resolved		Non/weak biofilm	Strong biofilm		Present	Not present	
S. aureus	I	15 (60)	10 (40)	0.1823	5 (20)	20 (80)	0.6629	1 (4)	24 (96)	0.3657
	II	2 (20)	8 (80)	0.0256*	2 (20)	8 (80)	0.8349	0 (0)	10 (100)	
	III	5 (71.4)	2 (28.6)	0.2419	1 (14.3)	6 (85.7)	0.7926	0 (0)	7 (100)	
	IV	1 (33.3)	2 (66.7)	0.5237	0 (0)	3 (100)		0 (0)	3 (100)	
S. epidermidis	I	15 (62.5)	9 (37.5)	0.7288	4 (16.7)	20 (83.3)	0.0129*	17 (70.8)	7 (29.2)	0.2399
	II	7 (70)	3 (30)	0.4705	1 (10)	9 (90)	0.0733	9 (90)	1 (10)	0.3057
	III	8 (50)	8 (50)	0.3221	12 (75)	4 (25)	<0.0001****	13 (81.2)	3 (18.8)	0.7035

a *P* values were statistically significant at *, *P* ≤ 0.05 and ****, *P* ≤ 0.0001 by chi-square test.

b Phenotypic multidrug resistance was considered when a strain was resistant to ≥3 antimicrobial agents.

### Phenotypic and genotypic properties of isolates.

There was a significant association between unresolved outcome and relapse caused by S. aureus (Table 6). The majority of relapses caused by S. epidermidis led to an unresolved outcome, whereas the other coagulase negative staphylococci (CoNS) species did not cause relapse.

**TABLE 6:** Association between bacterial virulence phenotype and clinical outcome in S. aureus and S. epidermidis isolates

Parameter	No. (%) of isolates		Odds ratio for not healed (95% confidence interval)	*P* value^*a*^
	Unresolved	Resolved		
S. aureus (*n *= 45)	23 (51.1)	22 (48.9)		
Biofilm formation			0 (0.00; 0.36)	0.0014***
Non/weak	0 (0)	8 (100)		
Strong	23 (62.2)	14 (37.8)		
Antibiotic resistance				
Methicillin				
Phenotypic AMR	0 (0)	3 (100)		
Genotypic AMR (*mecA* presence)	0 (0)	2 (100)		
Rifampicin				
Phenotypic AMR	0 (0)	0 (0)		
Multidrug resistance				
Phenotypic AMR	0 (0)	1 (100)		
Genotypic AMR	3 (75)	1 (25)	3.15 (0.43; 42.59)	0.3167
Relapse	11 (91.7)	1 (8.3)	19.25 (2.40; 215.60)	0.0010**
Reinfection	0 (0)	0 (0)		
S. epidermidis (*n *= 50)	30 (60)	20 (40)		
Biofilm formation			0.64 (0.21; 1.99)	0.4646
Non/weak	9 (52.9)	8 (47.1)		
Strong	21 (63.6)	12 (36.4)		
Antibiotic resistance				
Methicillin				
Phenotypic AMR	30 (65.2)	16 (34.8)	Infinity (1.54; infinity)	0.0107*
Genotypic AMR (*mecA* presence)	30 (65.2)	16 (34.8)	Infinity (1.54; infinity)	0.0107*
Rifampicin				
Phenotypic AMR	9 (90)	1 (10)	8.14 (1.25; 93.4)	0.0304*
Multidrug resistance				
Phenotypic AMR				
≥3 Antimicrobial agents	26 (66.7)	13 (33.3)	3.50 (0.81; 11.92)	0.0700
≥4 Antimicrobial agents	21 (75)	7 (25)	4.33 (1.261; 15.46)	0.0146*
≥5 Antimicrobial agents	16 (94.1)	1 (5.9)	21.71 (3.05; 238.6)	0.0004***
Genotypic AMR (≥3 genes)	19 (70.4)	8 (29.6)	2.59 (0.79; 7.96)	0.1049
Relapse	7 (77.8)	2 (22.2)	2.74 (0.61; 14.05)	0.2293
Reinfection	7 (87.5)	1 (12.5)	5.78 (0.84; 68.10)	0.083

a *P* values were statistically significant at *, *P* ≤ 0.05, **, *P* ≤ 0.01, ***, *P* ≤ 0.001 by chi-square test.

### Biofilm formation.

When all staphylococcal species were included, the biofilm production ability showed a trend where strains with strong biofilm ability were more frequent in unresolved PJI, and non- or weak biofilm strains were more frequent in resolved PJI (data not shown). When each species was evaluated separately, there was a significant positive correlation between strong biofilm production and unresolved PJI in S. aureus, whereas non/weak biofilm phenotypes of S. aureus were significantly associated with infection resolution (Table 6). All STs from S. aureus strains consisted mainly of strong biofilm producers (Table 3). Therefore, no statistical significance was observed to associate specific STs with biofilm formation in S. aureus.

No correlation was found in S. epidermidis between strong biofilm production and infection outcome (Table 6). However, in S. epidermidis, there were significant associations between a particular ST or *agr* type and biofilm formation. Specifically, ST2 strains (15/17 strains, *P *= 0.0172) and ST5 (9/10 strains, *P *= 0.0733) were strong biofilm producers, whereas ST215 (11/12 strains) were non/weak biofilm producers (*P < *0.0001) (Table 4). Thus, S. epidermidis *agr* I significantly correlated with strong biofilm production, while *agr* III significantly correlated with non/weak biofilm abilities (Table 5).

We then analyzed whether the EPS composition was related to the infection outcome. The results revealed that most of the S. aureus strains produced a biofilm matrix composed mainly of proteins (*n* = 20) or a combination of proteins and polysaccharides (*n* = 15) rather than polysaccharides alone (*n* = 1). Therefore, the EPS composition in S. aureus was not related to the infection outcome or biofilm production ability (Table 7; see Table S2 in the supplemental material). In contrast, for the S. epidermidis strains, the EPS was more commonly composed of polysaccharides, either alone (*n* = 11) or combined with proteins (*n* = 5), rather than with proteins alone (*n* = 2). In S. epidermidis, a trend was found between unresolved infection and polysaccharidic EPS (Table 7; see Table S3 in the supplemental material). Strong biofilm-producing S. epidermidis strains significantly more often contained polysaccharidic EPS than moderate biofilm producers, which contained a mixed EPS (proteinaceous and polysaccharidic) (Table 7).

**TABLE 7:** Association between extracellular polymeric substances (EPSs), clinical outcome, and biofilm formation in S. aureus and S. epidermidis isolates

Bacteria	Substance	No of isolates (%)		*P* value^*a*^	No of isolates (%)		*P* value^*a*^
		Unresolved	Resolved		Moderate^*b*^ biofilm	Strong^*c*^ biofilm	
S. aureus	Polysaccharide	1 (100)	0 (0)		0 (0)	1 (100)	
	Protein	12 (60)	8 (40)	0.5870	3 (15)	17 (85)	0.7642
	Polysaccharide and protein	10 (66.7)	5 (33.3)	0.7693	3 (20)	12 (80)	0.6501
S. epidermidis	Polysaccharide	9 (81.8)	2 (18.2)	0.0874	1 (9.1)	10 (90.9)	0.0012**
	Protein	0 (0)	2 (100)		2 (100)	0 (0)	
	Polysaccharide and protein	3 (60)	2 (40)	0.7098	4 (80)	1 (20)	0.0265*

a *P* values were statistically significant at *, *P* ≤ 0.05 and **, *P* ≤ 0.01 by chi-square test.

b Moderate biofilm production defined as 0.240 < optical density (OD) < 0.480.

c Strong biofilm production defined as OD of >0.480.

All S. aureus strains contained the biofilm-related genes *atl*, *clfA*, *sarA*, *spa*, *ica operon*, *arcA*, *agrA*, and *hld* (data not shown). The biofilm-associated genes found in the S. epidermidis strains are presented in Table 8 and Table S4 in the supplemental material. The presence of the *ica* operon and *aap* gene, important for the synthesis of polysaccharide and protein EPS components, respectively, was significantly higher in strains with strong biofilm production than in non/weak strains. The arginine deaminase gene *arcA*, important for survival at low pH in the biofilm maturation phase and inhibition of immune response, was found mainly in strong biofilm producers (54.5%) compared with non/weak producers (11.8%). When we further subdivided the biofilm-producing strains in moderate versus strong biofilm production, a higher presence of the *ica* operon was found in strong biofilm production than that in moderate production, but a higher presence of *bhp* was found in moderate biofilm production than that in strong production (Table S4).

**TABLE 8:** Association between biofilm-associated genes and biofilm-forming ability in S. epidermidis

Biofilm-associated gene in S. epidermidis	No. (%) isolates with gene and the indicated biofilm ability *in vitro*		*P* value^*a*^
	Non/weak (*n *= 17)	Strong (*n *= 33)	
*atlE*	17 (100)	33 (100)	
*embP*	17 (100)	33 (100)	
*ica* operon	2 (11.8)	19 (57.6)	0.0019**
*aap*	5 (29.4)	31 (93.9)	<0.0001****
*bhp*	4 (23.5)	12 (36.4)	0.3567
*arcA*	2 (11.8)	18 (54.5)	0.0034**

a *P* values were statistically significant at **, *P* ≤ 0.01 and ****, *P* ≤ 0.0001 by chi-square test.

### Antimicrobial susceptibility in planktonic (MIC) and biofilm (MBEC) conditions.

The evaluation of the phenotypic resistance of the 111 staphylococcal isolates using MIC susceptibility testing showed that only 1 S. aureus strain showed phenotypic multidrug resistance (strain 7270), and 4 other different S. aureus strains showed genotypic MDR (strains 6968, 6980, 7012, and 7013) (Fig. 1 and Table 6). Therefore, antibiotic resistance was not associated with treatment failure in S. aureus (Table 6 and 9). In the case of S. epidermidis, multidrug resistance as well as single resistance to rifampicin (RIF), oxacillin (OXA), or clindamycin (CLI) was significantly higher in strains from patients with treatment failure (Table 6 and 9). S. epidermidis strains of ST2 (16/17, *P* = 0.0483) were associated with phenotypic multidrug resistance (Table 4; Fig. 2). In addition, ST5 and ST215 showed high levels of phenotypic MDR. The majority of the RIF^r^ strains showed a strong biofilm phenotype (8/10), belonged mainly to the ST2 group (7/10), and contained the rep10 plasmid (8/10; GenBank accession no. GU562624), among others.

**TABLE 9:** Antibiograms of PJI strains for each tested antimicrobial agent according to MIC susceptibility testing

Antibiotic resistance^*a*^ by species	No. (%) of isolates		*P* value^*b*^
	Unresolved	Resolved	
S. aureus (*n *= 45)	23 (51.1)	22 (48.9)	
CIP	0 (0)	2 (100)	
CLI	0 (0)	1 (100)	
FA	1 (33.3)	2 (66.7)	0.5237
LZD	0 (0)	0 (0)	
OXA	0 (0)	3 (100)	
RIF	0 (0)	0 (0)	
TRI/SUL	0 (0)	0 (0)	
VAN	0 (0)	0 (0)	
S. epidermidis (*n *= 50)	30 (60)	20 (40)	
CIP	26 (65)	14 (35)	0.1489
CLI	23 (74.2)	8 (25.8)	0.0089**
FA	20 (71.4)	8 (28.6)	0.0627
LZD	0 (0)	0 (0)	
OXA	30 (65.2)	16 (34.8)	0.0107*
RIF	9 (90)	1 (10)	0.0304*
TRI/SUL	17 (68)	8 (32)	0.2482
VAN	0 (0)	0 (0)	

a CIP, ciprofloxacin; CLI, clindamycin; FA, fusidic acid; LZD, linezolid; OXA, oxacillin + 2% NaCl; RIF, rifampicin; TRI/SUL, trimethoprim-sulfamethoxazole; VAN, vancomycin.

b *P* values were statistically significant at *, *P *≤ 0.05 and **, *P *≤ 0.01 statistically significant by chi-square test.

**FIG 3:**
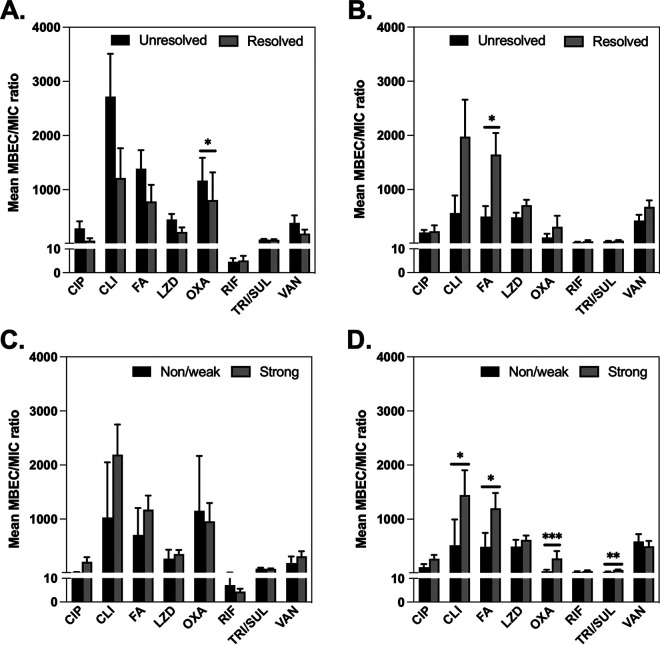
MBEC/MIC ratios in relation to clinical infection outcome for S. aureus (A) and S. epidermidis (B) and the relation to biofilm formation for S. aureus (C) and S. epidermidis (D). Data are represented as means with SD. *, *P ≤ *0.05; **, *P* ≤ 0.01; ***, *P ≤ *0.001; statistically significant, Mann-Whitney test. MBEC, minimum biofilm eradication concentration, CIP, ciprofloxacin; CLI, clindamycin; FA, fusidic acid; LZD, linezolid; OXA, oxacillin + 2% NaCl; RIF, rifampicin; TRI/SUL, trimethoprim-sulfamethoxazole; VAN, vancomycin, biofilm production (non/weak versus strong).

The minimum biofilm eradication concentration (MBEC)/MIC ratios represent the fold increase in antimicrobial concentration required to inhibit or kill the strain when grown as a biofilm compared with those as planktonic growth and were calculated by dividing the MBEC value by the MIC value. MBEC/MIC ratios were compared to evaluate the increased tolerance to antimicrobials of the strains when grown as biofilms *in vitro*. In strains from nonresolved infections, the lowest and highest mean MBEC/MIC ratios for S. aureus ranged between 5 (RIF) and 2,718 (CLI), and for S. epidermidis, they ranged between 26 (RIF) and 565 (CLI). The S. aureus strains in unresolved infections had a significantly increased OXA ratio (median ratio, 1,166) compared with that in resolved infections (median ratio, 808) (*P ≤ *0.05) (Fig. 3A); however, no association was found between S. aureus MBEC/MIC ratios and biofilm production (Fig. 3C). The S. epidermidis strains in the unresolved group had significantly lower fusidic acid (FA) MBEC/MIC ratios (1,645) than the strains from patients with resolved outcomes (500) (Fig. 3B). Strong biofilm-producing S. epidermidis strains showed increased biofilm resistance (MBEC/MIC ratio) to CLI, FA, OXA, and trimethoprim-sulfamethoxazole (TRI/SUL) compared with non/weak strains (Fig. 3D).

**FIG 4:**
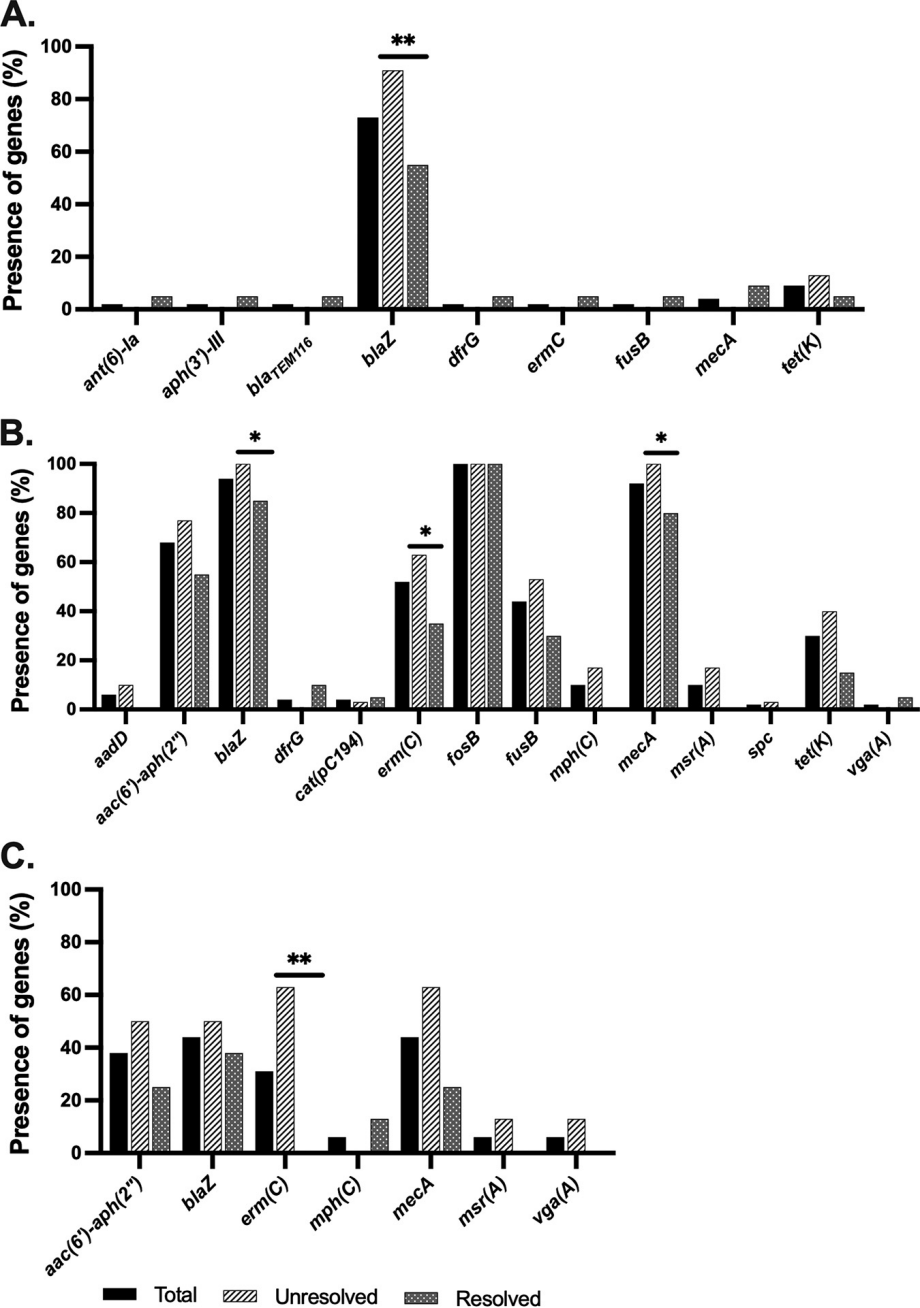
Percentages of antimicrobial resistance genes present in S. aureus (A), S. epidermidis (B), and other CoNS (C) in the two infection outcome groups (unresolved and resolved). Black bars, “total”; hatched white bars, “unresolved” outcome; hatched gray bars, “resolved” outcome. *, *P ≤ *0.05; **, *P ≤ *0.01; statistically significant, chi-square test.

The relative presence of antimicrobial resistance genes was evaluated in both patient outcome groups. The S. aureus and S. epidermidis strains from unresolved outcome patients contained a significantly higher presence of *blaZ* than strains from patients with resolved outcomes (Fig. 4A and B). Furthermore, the plasmid rep16 (GQ900401) carrying the *blaZ* gene was significantly more abundant in S. aureus strains from unresolved infections (64.5%) than in those from resolved infections (35.5%) (see Table S5 in the supplemental material).

All the S. epidermidis strains in the unresolved outcome that were resistant to OXA contained both *mecA* and *blaZ* resistance genes. Overall, the level of OXA_r_ in the majority of S. epidermidis was high, with only 4/50 OXA_s_, of which two contained *lacZ*. In addition, a significantly higher percentage of S. epidermidis strains from patients with unresolved infection carried the *blaZ*, *erm(C)*, and *mecA* genes than those from resolved infection (Fig. 4B). In addition, *erm(C)* was significantly more present in unresolved infection caused by other CoNS than in resolved infection (Fig. 4C).

### Carriage of other virulence factors.

**FIG 5:**
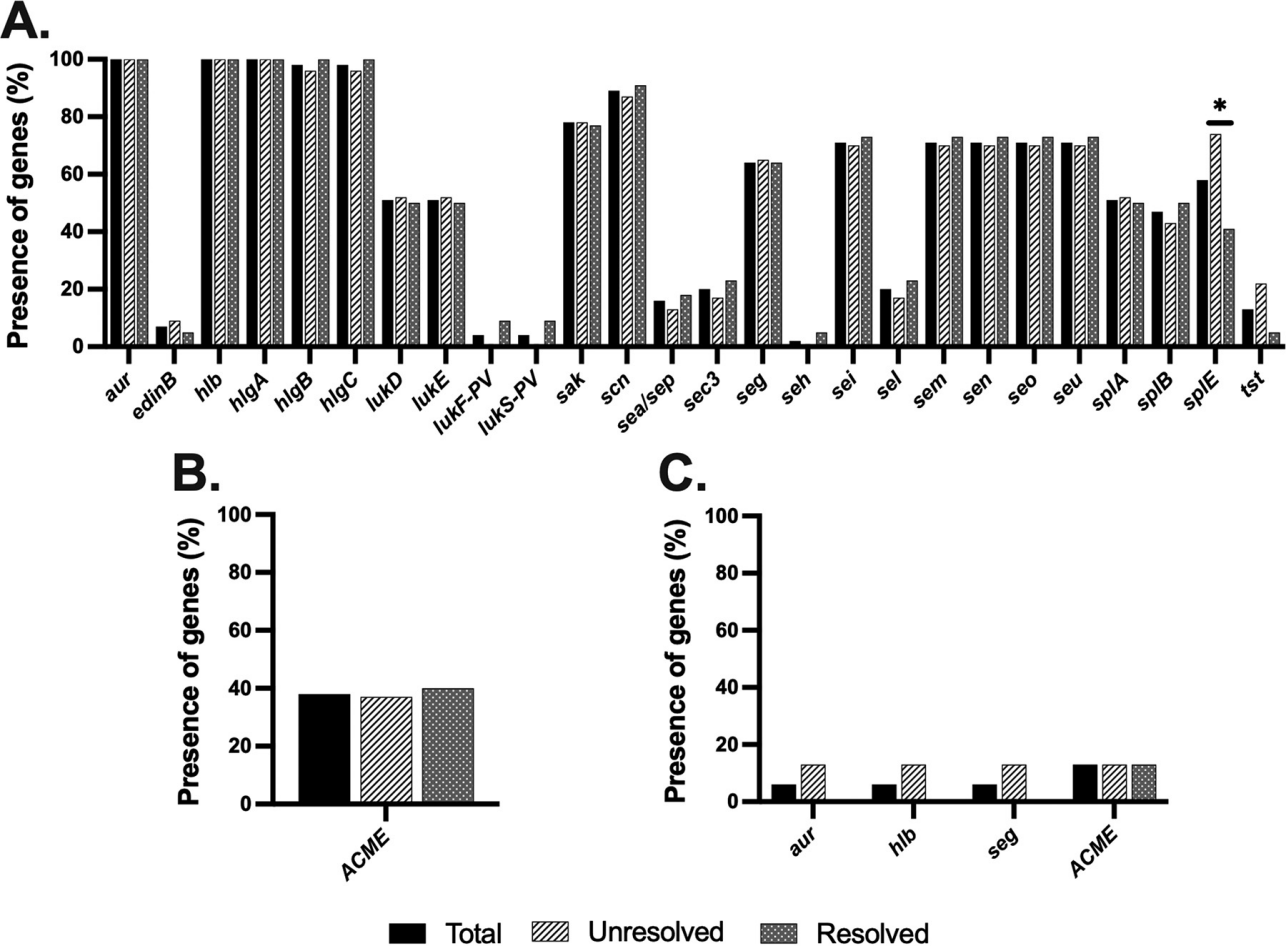
Percentages of virulence genes present in S. aureus (A), S. epidermidis (B), and other CoNS (C) in the two infection outcome groups (unresolved and resolved). Black bars, “total”; hatched white bars, “unresolved” outcome; hatched gray bars, “resolved” outcome. *, *P ≤ *0.05; statistically significant, chi-square test.

A wide array of virulence genes was identified and compared among the outcome groups. Only the serine protease gene *splE* was significantly more frequent in S. aureus from unresolved infection than resolved (Fig. 5A). The toxic shock syndrome toxin-1 gene *tst*, although not significant, was more frequently found in S. aureus from unresolved infections. A relatively high proportion of S. epidermidis strains (38%) were positive for the arginine catabolic mobile element (ACME); however, it was not associated with patient outcome (Fig. 5B). For the other CoNS, a few virulence genes were detected in a low proportion (Fig. 5C).

## DISCUSSION

In this study, we evaluated the relationship between the genomic features of staphylococcal strains isolated from patients with PJI and their capacity to produce biofilms, antimicrobial susceptibility, and clinical outcomes. We examined staphylococcal isolates retrospectively collected from 66 patients with PJI in Sweden and followed up with the patients for up to 5 years.

Interestingly, we found that S. aureus with strong biofilm abilities is closely linked to unresolved infection outcomes, whereas S. epidermidis resistance to antibiotics (i.e., RIF^r^, methicillin resistance, and MDR) plays a major role in unresolved infection outcomes. When we studied the EPS composition in S. aureus, it seemed to not be related to the patient outcome or the biofilm production ability. Even though the biofilm formation ability in S. epidermidis did not correlate with patient outcome, the biochemical nature of the EPS could play a role. Although not statistically significant, S. epidermidis strains producing polysaccharidic EPS more often caused unresolved infection (in 81.8% of cases), and those that produced only proteinaceous EPS were found in patients with a resolved outcome. Moreover, strong biofilm-producing strains contained more polysaccharidic EPS than moderate strains, which contained a mixed EPS. This result was further confirmed by the higher presence of the polysaccharidic factor *ica* operon with stronger biofilm production, while a higher presence of the proteinaceous factor *bhp* was found in moderate biofilm producers.

Overall, S. epidermidis strains with phenotypic and genotypic antibiotic resistance were highly present in patients with treatment failure of PJI, especially strains with phenotypic resistance to ≥4 antimicrobial agents significantly associated with unresolved infection. A similarly high prevalence of MDR has been reported previously in S. epidermidis from PJI in another geographical region of Sweden (28, 29). These findings further suggest that the current treatment strategies of PJIs may be selecting for nosocomial MDR S. epidermidis lineages through a horizontal gene transfer of AMR genes into various genetic backgrounds (29). The mean MIC^VAN^ was 1.8 μg/mL (range, 1 to 4) for all staphylococcal strains; therefore, no vancomycin-intermediate-resistant staphylococci were detected that could have influenced treatment outcomes. Resistance to rifampicin was significantly higher in strains from patients with treatment failure, supporting the results from previous studies where inadequate rifampicin regiments lead to worse outcomes (30). Even though rifampicin resistance (RIF_r_) was among the lowest, 20% (*n* = 10) of the S. epidermidis strains were RIF_r_, of which 9/10 strains were associated with treatment failure. As these isolates were not all from primary infections, they could have emerged after long-term treatment with RIF of the primary/earlier infection. All these results have major clinical implications since the combination of rifampicin with a second antibiotic is the first line of therapy in PJI due to its antibiofilm activity, putting additional pressure on thorough surgical debridement procedures and the choice of best second-line antimicrobial agents. In this regard, the characterization of biofilm abilities and MBEC susceptibility testing would be relevant clinical diagnostic tools (31) since biofilm formation and antibiotic resistance have been associated with poor outcome in S. epidermidis orthopedic-device-related infections (32).

Previously, the *blaZ* gene has been found frequently in methicillin-susceptible S. aureus isolates causing PJI (33). However, in the present study, we have additionally shown that *blaZ* in S. aureus and S. epidermidis strains was detected more frequently in unresolved infections than in resolved infections. Interestingly, all S. epidermidis strains in the patient group with unresolved infection were 65.2% resistant to OXA, and all contained *mecA* and *blaZ* genes. A few reports have shown that orthopedic patients, health care workers, and hospital environments carry methicillin-resistant S. epidermidis (MRSE) (34–36). A significant prevalence of methicillin resistance and carriage of the *mecA* gene (92%) was found in the S. epidermidis strains from this study, which is higher than the 80% prevalence found by Hellmark et al. in S. epidermidis PJI isolates (28). This finding supports the previous indication that methicillin resistance in clinical S. epidermidis isolates is increasing in health care settings (37). Moreover, in this study, MRSE strains were associated with treatment failure, supporting previous observations that patients with MRSE infections had significantly lower healing rates than patients with infections caused by methicillin-susceptible S. epidermidis (38).

All staphylococcal strains showed a significant increase in antibiotic resistance when grown *in vitro* as biofilms (MBECs) compared with planktonic cultures (MICs), reaching MBEC/MIC ratios up to 8,192, with the lowest ratios for RIF. In the S. aureus strains, biofilm production and its associated increased tolerance to antimicrobials may have been the main virulence factor causing treatment failure since genetic and phenotypic antimicrobial resistance in S. aureus strains was generally low. In contrast, Wildeman et al. found that an antibiotic-resistant phenotype in S. aureus was associated with failure in hospitals from two different regions of Sweden (39); however, the biofilm-forming ability of the strains was not evaluated. In the present study, S. epidermidis antibiotic resistance and not biofilm formation correlated with treatment failure. Conversely, Morgenstern et al. found that biofilm-forming ability in S. epidermidis from ODRI influenced treatment outcome, whereas antimicrobial resistance did not. Infections caused by CoNS often needed second-line antimicrobial agents due to broad antimicrobial resistance in health care settings (40). For example, in PJIs, the high overall S. epidermidis resistance to fluoroquinolones (41) likely impacts overall outcomes negatively, as the fluoroquinolone-rifampicin combination is currently the favored treatment (42). It has been suggested that MDR in S. epidermidis is probably due to the accumulation of resistance genes and to high antibiotic pressure in hospital care, which together with the acquisition of biofilm-associated genes give a selection advantage to invasive isolates (28).

A broad array of virulence genes was detected in the S. aureus PJI strains; however, only the serine protease *splE* gene was significantly associated with unresolved infection. Out of 26 *splE*-positive S. aureus, 17 were found in unresolved infections, and 23 were strong biofilm producers. Although not significant, all but 1 (5/6) S. aureus strain containing the toxic shock syndrome toxin-1 gene (*tst*) was associated with unresolved infection and belonged to ST30-*agr* III and were strong biofilm producers. The *tst* gene has been proposed to play a role in infection pathogenesis via direct and persistent toxic functions and by increasing the secretion of inflammatory cytokines that indirectly induce immune suppression (43).

The arginine catabolic mobile element (ACME) in S. epidermidis was relatively high (38%) compared with 13% found in a previous study (28). The presence of ACME among S. epidermidis isolates is considered an important virulence factor by virtue of its capacity for staphylococcal cell colonization of the host (human skin, mucosal surfaces, and indwelling medical devices) (44). The majority of ACME-positive strains were strong biofilm producers, whereas the majority of ACME-negative strains were non/weak biofilm producers. All ACME-positive strains were also positive for *arcA*, as the *arc* operon is associated with the ACME genetic island, in addition to the chromosomal *arc* (44).

Few studies have focused on correlating the staphylococcal genome with patient outcome. A previous study did not find an association between genomic traits in S. aureus and patient outcome (39). In this study, the various STs found in S. aureus did not correlate with unresolved outcomes. Furthermore, ST45 was the most prevalent S. aureus clone, as reported previously (45). The global MDR S. epidermidis lineages ST2 and ST5 and regional ST215 have been found to be common in S. epidermidis from PJI and other infections in Sweden (28, 29, 46). These common S. epidermidis STs corresponded strictly to specific *agr* types (ST2 with *agr* I, ST5 with *agr* II, and ST215 with *agr* III), as described previously (47). S. epidermidis ST2 was significantly associated with MDR and strong biofilm production, and ST215 was associated with MDR and non/weak biofilm production. The association between *agr* groups and biofilm formation has been studied widely in S. aureus; however, less is known about S. epidermidis. In S. epidermidis, *agr* I strains were significantly associated with strong biofilm production (*ica* positive), while *agr* III strains were associated with non/weak biofilm production.

Systemic disease according to the ASA classification was associated significantly by univariate analysis with unresolved infection, extending previous conclusions that patients with ASA of ≥3 have a higher risk for persistent infection (39). Patients with an unresolved infection commonly experienced 2 to 6 reoperations. Revision surgery patients have a higher risk of infection than with primary surgery since revision surgery implies retraumatizing the infected implant-tissue area, increasing the risk of disseminating bacteria by debridement procedures, tissue loss, and poorly perfused tissue defects (48). The results from this study indicate that high CRP values (>130 mg/L) at the diagnosis of PJI may be indicative of failed treatment, which is supported by other studies (49). Furthermore, Xu et al. addressed the need to reexamine the current thresholds for acute PJI since low-virulent bacteria like CoNS could lead to false-negative CRP (50). Indeed, this study further confirmed that CRP values were significantly higher in patients infected with S. aureus than those of patients infected by S. epidermidis. Although the absolute CRP values were lower, a similar 3-fold increase in synovial fluid CRP from PJI patients has been reported previously (51). Even though S. aureus is well known to carry a wide array of virulence factors causing high tissue damage and immune evasion, the patient outcome did not seem to be influenced by the type of staphylococcal species causing the infection. Instead, the ability of S. aureus to form biofilm played a more significant role in the infection outcome, associating with unresolved infection.

Among the limitations of the study is the retrospective cohort design since not all strains isolated from the patients were stored. When several strains were coisolated from the same patient, all strains were analyzed as the causative agent, as any synergistic effect between the strains is unknown. Our analysis model allocated all strains of a polymicrobial infection to the same clinical outcome. Unfortunately, due to the limited number of samples, we could not have enough statistical power to ascertain some associations, especially regarding the MLSTs and infection outcome. Prospective studies are warranted to guarantee that all isolated strains are evaluated in regard to future relapses and to fully correlate their genetic traits with the patient’s infection outcome.

In conclusion, strong biofilm production in S. aureus and antimicrobial resistance in S. epidermidis significantly correlated with unresolved PJIs. These traits should be considered important risk factors for the diagnosis and guidance of clinical treatment in PJI. The clinical relevance of some particular genetic variants and virulence factors associated with treatment failure of PJI remains unknown, and further studies need to be conducted to ascertain their prevalence and role in PJI pathogenesis and as prognostic factors.

## MATERIALS AND METHODS

### Study population, clinical parameters, and outcome.

The study protocol was approved by the Regional Ethics Review Board of Gothenburg (Sweden) (Dnr 654-16), and written consent was obtained from patients. This retrospective study included patients admitted at the Sahlgrenska University Hospital (Mölndal, Sweden) with PJI of the hip and knee between 1 January 2012 and 30 June 2015, with a follow-up period between 3.5 and 5 years (31 December 2018). A total of 66 patients (subjected to 43 DAIR, 29 revisions, and 9 other procedures) and 111 staphylococcal isolates from intraoperative tissue samples, which were saved and stored at the Clinical Bacteriological Laboratory at Sahlgrenska University Hospital, were included in the study. A detailed clinical and phenotypic investigation on biofilm antimicrobial resistance on a subset of these isolates (70 strains from first-time infections) has been described previously; however, no genomic data were included (31). The inclusion criteria were as follows: PJI of total hip or knee arthroplasty using the MSIS 2018 criteria (52) and monomicrobial infection caused by either Staphylococcus aureus or coagulase negative staphylococci (CoNS) or polymicrobial infection by two staphylococcal species.

The primary aim was to evaluate the correlation of staphylococcal genome variation and carriage of virulence factors to clinical outcome (resolved versus unresolved infection) with biofilm-forming ability or susceptibility (antibiograms and MBEC/MIC ratios) and clinical outcome.

A resolved infection was defined as no suspicion of infection (clinical or laboratory) and no further treatment (surgical or antimicrobial) due to PJI within a follow-up period between 3.5 and 5 years. An unresolved infection was either due to relapse or reinfection. Relapse is an infectious recidivate due to the failed treatment of the original strain, while reinfection is the isolation of a different bacterial strain than the one that originated the previous infection. Relapses were confirmed by core-genome multilocus sequence typing (cgMLST), MLST type, and antimicrobial susceptibility pattern.

### DNA isolation, genome sequencing, and bioinformatic analyses.

The 111 strains were grown on tryptic soya agar (TSA) overnight (o.n.) at 37°C. One colony was inoculated on 5 mL tryptic soy broth (TSB) + glucose and incubated at 37°C and 200 rpm. A volume of 1.5 mL of the culture was centrifuged at 16,000 × *g* for 2 min, and genomic DNA was isolated from the pellet using the GenElute bacterial genomic DNA kit (Sigma-Aldrich, St. Louis, MO, USA) following the manufacturer’s instructions for Gram-positive bacteria, including the initial lysing step with Gram-positive lysis solution supplemented with lysozyme (50 mg/mL) and lysostaphin (0.0665 mg/mL) and the optional RNase A treatment to remove residual RNA. The DNA was eluted in elution buffer (10 mM Tris-HCl, pH 8.5) and stored at −20°C. The concentration and quality of the genomic double-stranded DNA (dsDNA) was assessed by gel electrophoresis (0.8% agarose) and in a nanophotometer (Thermo NanoDrop 2000; Thermo Scientific). The purified DNA samples were sequenced at MicrobesNG (http://www.microbesng.uk) using the Nextera XT library and a HiSeq 2500 Illumina sequencer, removing GC coverage bias for AT-rich genomes, such as Staphylococcus spp., and a minimum of 30× coverage. Reads were adapter trimmed using Trimmomatic 0.30 with a sliding window quality cutoff of Q15 (53). De novo assembly was performed on samples using SPAdes version 3.7 (54), and contigs were annotated using Prokka 1.11 (55). The species classification was further confirmed by taxonomic distribution analysis using the bioinformatic tool Kraken (56).

Using publicly available bioinformatic tools from the Center for Genomic Epidemiology (CGE) (Technical University of Denmark) (57) and its Bacterium Analysis Pipeline (BAP), which is a single pipeline for batch uploading of whole-genome sequencing (WGS) data from multiple isolates, the following parameters were identified: bacterial species, assembly of the genome (draft *de novo* assembly of sequencing reads into contigs), multilocus sequence type (MLST), plasmids, virulence genes, and antimicrobial resistance genes.

The *agr* types of S. aureus and S. epidermidis were downloaded from NCBI (https://www.ncbi.nlm.nih.gov/). For S. aureus, the GenBank identifiers (IDs) M21854.1 (Agr 1), AF001782.1 (Agr 2), AF001783.1 (Agr 3), and AF288215.1 (Agr 4) were used.

For S. epidermidis, the GenBank IDs Z49220.1 (Agr 1), AF346724.1 (Agr 2), and AF346725.1 (Agr 3) were used. Each contig was converted to a database with blast+ (2.7.1) (58). Nucleotide Basic Local Alignment Search Tool (BLASTn) searches were performed using the *agr* types as queries and the contigs as subjects. The BLAST hit with the highest percentage identity for each sample was used to define the *agr* class. In addition, BLASTn searches were performed to identify the presence of biofilm-associated genes in S. aureus and S. epidermidis. Ninety percent identity was used as the threshold, and S. aureus NCTC 8325 (NC_007795.1) and S. epidermidis ATCC 12228 (NC_004461.1) were used as reference genomes for all the genes. Information on the presence of the *ica* operon (*icaA*, *icaB*, *icaC*, *icaD*, and *icaR*) was extracted from the supplied annotation files.

### Core-genome multilocus sequence typing (cgMLST) and phylogenies.

The 1928 platform (1928 Diagnostics, Gothenburg, Sweden) was used to perform core-genome multilocus sequence typing (cgMLST) analysis of the S. aureus and S. epidermidis strains. The cgMLST typing identifies the specific core gene alleles of a particular strain and how many of the core genes differ between a set of strains. This information is then used to create a phylogenetic tree using the unweighted pair group method with arithmetic mean (UPGMA) method.

The cgMLST scheme for S. aureus did already exist, but a cgMLST scheme for S. epidermidis was created for this study and was added to the platform. To create the scheme, the genome of S. epidermidis with RefSeq assembly number GCF_009685135.1_ASM968513v1 was used to select targets for candidate core genes. S. epidermidis complete genomes available from NCBI RefSeq that were available on the 8 February 2022 (*n* = 87) were used as reference genomes, and the resulting cgMLST scheme contained genes found in 95% of reference genomes under an identity threshold of 90%. The resulting scheme was comprised of 1,683 coding genes.

To validate the generated cgMLST scheme, a random selection of 300 samples from all the available S. epidermidis samples on the European Nucleotide Archive (ENA) (*n* = 5,311) were chosen and ran within the 1928 platform. Out of the 300 samples, 8 samples failed due to intraspecies contamination as detected by the 1928 platform. An additional 14 samples failed due to low sequencing depth (<30×), and 7 samples were not confidently identified as S. epidermidis by the 1928 platform. From the remaining 271 samples, 21 (7.75%) samples produced cgMLST results where the fraction of core genes present to the total number of core genes in the scheme (fraction of core) was below 95%. Out of the 21 samples with low fraction of core, 18 belonged to MLSTs for which other samples had a high fraction of core. This finding suggests a poor performance of these samples as a result of uneven sequencing depth and that the scheme is robust for the different phylogenetic clades of the species. The 271 samples represented a diverse set of 65 MLSTs.

### Categorization of biofilm-formation ability and analysis of EPS composition.

To categorize the clinical strains according to their biofilm-forming abilities *in vitro*, the microtiter plate test was performed using a previously described protocol (31, 59). In brief, for each clinical strain, one colony was cultured in tryptic soy broth (TSB) (+0.25% glucose for S. aureus) at 37°C and 200 rpm. The culture was adjusted to an optical density at 546 nm (OD_546_) of 1 and 1:40 diluted in TSB (+Glu), and 200 μL was dispensed in triplicate wells (BioLite cell culture treated plates; Thermo Scientific, MA) and incubated for 24 h at 37°C. Wells were rinsed 3 times in water, stained with crystal violet (2%; VWR, PA), washed 3 times, air dried, and eluted in ethanol-acetone (80:20, vol/vol) for 5 min, and 150 μL was transferred to a new plate to measure the OD_595_ with a plate reader (FLUOstar Omega; BMG Labtech, Offenburg, Germany). Sterile TSB (+Glu) was included as a blank. The mean of the blank corrected values served to categorize the strains into biofilm-forming categories using breakpoints from Baldassarri et al., as follows: (60) nonproducer (OD, <0.120), weak producer (0.120 < OD < 0.240), and strong producer (OD, >0.240). For the statistical analysis and for a similar distribution among groups, biofilm-forming categories were dichotomized to (i) non/weak producer (OD, <0.240) and (ii) strong producer (OD, >0.240). The control strains S. epidermidis ATCC 35984 and ATCC 12228 and S. aureus 15981 and 15981 Δ*ica* for strong and nonbiofilm production, respectively, were included.

Thereafter, biofilm-producing strains (OD, >0.240) were further categorized according to their biofilm extracellular polymeric substances (EPSs) produced *in vitro* following a previously described biofilm detachment assay (61). In brief, biofilms were grown for 24 h on polystyrene microtiter plates as described above and treated either with 100 μL dispersin B (40 μg/mL; Kane Biotech Inc., MB, Canada) or with 100 μL proteinase K (100 μg/mL; Sigma-Aldrich) for 2 h at 37°C (duplicate wells). Untreated biofilms (1% phosphate-buffered saline) were included as a control. After incubation, the wells were emptied, gently washed 3 times by immersion in water, and air dried. Crystal violet staining of biofilms was then performed as described above. Biofilms sensitive to detachment by proteinase K suggested an EPS composed of proteins, whereas biofilms sensitive to detachment by dispersin B had an EPS composed of polysaccharides. Biofilms detached by both treatments showed a mixed EPS composition of both polysaccharides and proteins. Only strains with a statistically significant reduction in crystal violet OD_595_ by dispersin B or proteinase K versus untreated control were classified under these three EPS categories. The following control strains were included: S. aureus 15981 wild type (WT) and 15981 Δ*ica* as a positive and negative control, respectively, for polysaccharide (PIA) production and V329 WT and V329 Δbap as a positive and negative control, respectively, for protein (Bap) production.

### Antimicrobial susceptibility testing.

The broth microdilution method was performed to determine the MIC and minimum biofilm eradication concentration (MBEC) of the 111 strains toward doubling increasing concentrations (0.25 to 1,216 μg/mL) of eight antimicrobial agents used in the treatment of PJI, as follows: ciprofloxacin (CIP), clindamycin (CLI), oxacillin + 2% NaCl (OXA), fusidic acid (FA), linezolid (LZD), rifampicin (RIF), trimethoprim-sulfamethoxazole (SXT), and vancomycin (VAN). The MIC and MBEC methods have been described in detail previously (31, 62). The Calgary Biofilm Device (MBEC assay; Innovotech Inc., Edmonton, Canada) and a custom-made broth microdilution plate (CML2FNUN; Sensititre, Thermo Scientific, MA) were combined to determine the MBEC of the strains. MIC determinations were performed on planktonic cultures of all strains with equal final concentrations for the MBEC determination. The S. aureus ATCC 29213 strain was used as a control. When there was growth in the well with the highest concentration, the next virtual 2-fold increase was chosen for calculations.

The strains were categorized as susceptible or resistant as follows: phenotypic antimicrobial resistance (AMR) was assessed according to EUCAST MIC breakpoints for the eight antimicrobial agents (63) and genotypic AMR was assessed according to the presence of AMR genes for a total of 11 antimicrobial agents (57). Phenotypic multidrug resistance (MDR) was considered when a strain was resistant to ≥3 antimicrobial agents, although MDR to ≥4 and ≥5 antimicrobial agents was also evaluated. Genotypic MDR was considered when a strain was resistant to ≥3 AMR genes. Phenotypic methicillin resistance was screened by OXA MIC and genotypically confirmed by the presence of the *mecA* gene.

### Statistical methods.

All data were stored in SPSS version 27 and Microsoft Excel. Furthermore, all statistical analyses and graph customizations were carried out using GraphPad Prism 9. Associations between clinical parameters were analyzed using the chi-square test. A *t* test was used to analyze significant differences between two groups’ means. Interquartile ranges were used to show the spread of inflammatory markers in all patients. The Mann-Whitney test was used to compare the MBE/MIC ratios of strains from infection outcome groups and from biofilm categories. The presence or absence of ST and *agr* types, AMR and virulence genes and phenotypes, and EPS composition among the infection outcome groups, biofilm categories, and MDR were analyzed using the chi-square test. Statistical significance is represented as following: *, *P* ≤ 0.05; **, *P* ≤ 0.01; ***, *P* ≤ 0.001; and ****, *P* ≤ 0.0001.

### Data availability.

All sequenced genomes are available as BioProject PRJNA765573 in NCBI.
