# Supplementary material for: Genomics of Staphylococcus aureus and Staphylococcus epidermidis from Periprosthetic Joint Infections and Correlation to Clinical Outcome
Source: Microbiol Spectr. 2022 Jun 28;10(4):e02181-21. doi: 10.1128/spectrum.02181-21 (PMC9430453; doi:10.1128/spectrum.02181-21)
Supplement: Supplemental file 1 — Supplemental material. Download spectrum.02181-21-s0001.pdf, PDF file, 0.09 MB [file spectrum.02181-21-s0001.pdf]

Supplementary Materials

Table S1 Demography of study cohort including inflammatory markers and antimicrobial treatment

Baseline demographics (total patients = 66)	
**Sex** *n (%)*	
Male	44 (66.7)
Female	22 (33.3)
**Age** *mean years (SD)*	70 (13)
**BMI** *mean (SD)*	28.7 (5.6)
**Obesity**[a] *n (%)*	24 (36.4)
**Smoking** *n (%)*	4 (6.1)
**Diabetes** *n (%)*	11 (16.7)
**Sepsis** *n (%)*	3 (4.5)
**ASA class** *n (%)*	
1: Healthy	4 (6.1)
2: Mild systemic disease	38 (57.6)
3, 4: Severe systemic disease, potential life threat	23 (34.8)
Note: missing data for 1 patient	
**Arthoplasty type** *n (%)*	
THA	46 (69.7)
TKA	21 (31.8)
Note: 1 patient receive both hip and knee surgery	
Inflammatory markers *median (IQR)*	
ESR	46 (23-82)
WBC	7.9 (6.1-10)
CRP	50 (22-163)
Antimicrobial treatment	
**Antimicrobial agent iv** *n (%)*	
VAN or VAN/poly	45 (68.1)
Other	20 (30.3)
Missing	2 (3.03)
Note: 1 patient received VAN and another antimicrobial agent	
**Antimicrobial oral** *n (%)*	
RIF/poly	40 (60.6)
Other	23 (34.8)
None	5 (7.6)
Missing	2 (3.0)
Note: 4 patients received RIF/poly and other antimicrobial agents	
**Duration of therapy (iv and oral)** *mean weeks (SD)*	15.2 (8.8)
Recurrent Infection	
**Relapse** *n (%)*	10 (15.2)
**Reinfection** *n (%)*	9 (13.6)
Clinical infection Outcome	
**Resolved** *n (%)*	35 (53.0)
**Unresolved** *n (%)*	31 (47.0)

[a]Defined as a BMI of >30. Abbreviations: SD = standard deviation, IQR = interquartile range, BMI = body mass index, ASA = American Society of Anesthiologists Physical status, THA = total hip arthroplasty, TKA = total knee arthroplasty, ESR = erythrocyte sedimentation rate, WBC = white blood cell, CRP = C-reactive protein, iv = intravenous, VAN/poly = vancomycin in combination with other intravenous antimicrobial agent, RIF/poly = rifampicin in combination with other oral antimicrobial agent.

Table S2 Composition of extracellular polymeric substances (EPS) in the biofilms formed by *S. aureus* from PJI

Isolate	% OD reduction to control		Isolate	% OD reduction to control	
	Dispersin B	Proteinase K		Dispersin B	Proteinase K
6920	39.0	68.7	6950	76.7***	219.3****
6977	90.1**	113.1**	6958	18.3**	58.7***
7012	44.5	145.4**	6968	-8.6	114.5***
6921	96.8*	109.4**	6980	92.0***	118.4***
6924	36.7	134.9**	6990	33.4**	48.5**
6927	30.1*	69.5**	6992	27.2*	32.8*
6979	41.5*	97.9**	7004	50.2*	92.4***
7013	-11.0	60.5	7011	1.6	14.7*
6922	24.1	121.0*	7015	-7.9	20.0*
6936	13.2*	25.2**	6982	23.6	65.4*
6937	12.1	45.2	7002	2.4	55.0*
6943	71.9**	18.0	7009	28.3	76.0**
6948	46.3	123.6	7014	55.9*	104.2*
6953	17.4	44.1	7018	28.8	39.6
6963	52.5	89.4	7027	64.0*	110.5**
6974	34.2	92.4*	7031	35.5	92.3**
6942	59.6	194.4**	7022	22.1	27.5
6947	73.4*	177.0**	7028	39.1	58.8*

Note: *$P \leq 0.05$, **$P \leq 0.01$, ***$P \leq 0.001$, ****$P \leq 0.0001$ statistically significant, unpaired *t* test. Significant reduction in crystal violet OD after dispensin B treatment represents EPS mainly composed of polysaccharides whereas significant reduction in crystal violet OD after proteinase K treatment represents EPS mainly composed of proteins.

Table S3 Composition of extracellular polymeric substances (EPS) in the biofilms formed by *S. epidermidis* from PJI

Isolate	% OD reduction to control		Isolate	% OD reduction to control	
	Dispersin B	Proteinase K		Dispersin B	Proteinase K
6925	-1.5	10.7**	6923	94.8***	-8.1
6928	1.9	-21.4	6932	18.9	59.5*
6933	15.4	22.6*	6940	-5.9	-17.2
6951	-5.8	-16.7	6946	84.0*	32.8
6959	-1.2	-1.1	6955	6.6	10.3*
6926	47.3*	46.9*	6957	-4.5	63.6**
6929	6.1*	15.7**	6962	85.5***	-22.2
6938	0.2	1.0	6965	4.0	3.4*
6969	-0.2	15.8*	6994	298.5****	-0.1
6983	0.8	-10.7	6960	42.6*	-19.1
6985	53.5*	39.1*	6964	41.1***	7.3*
6987	0.2	-8.6	6978	61.0*	1.7
7003	14.1**	5.0	6995	319.2****	-0.1
7008	2.1	-6.2	6996	310.7****	0.4
6984	49.6*	23.7	7007	282.8***	-9.6
6986	1.9	-1.6	7017	69.1**	43.5*
6988	1.8	-9.9	7023	-26.6	-23.1
7005	40.9***	10.9**	6944	31.7**	31.0**
7021	31.4**	37.6**	7006	316.6****	50.3
7025	12.7*	26.9*	7010	118.0**	5.7
6930	-1.0	-8.5	7020	18.4	2.3
6939	-5.9	-12.8	7024	26.0*	4.4
6949	-5.2	17.5**			

Note: *$P \leq 0.05$, **$P \leq 0.01$, ***$P \leq 0.001$, ****$P \leq 0.0001$ statistically significant, unpaired *t* test. Significant reduction in crystal violet OD after dispensin B treatment represents EPS mainly composed of polysaccharides whereas significant reduction in crystal violet OD after proteinase K treatment represents EPS mainly composed of proteins.

Table S4 Distribution of biofilm associated genes and biofilm-forming ability in *S. epidermidis*

**Biofilm associated gene in *S. epidermidis***	**No. (%) isolates with gene and the indicated biofilm strength *in vitro***			
	**Total (*n* = 50)**			
	**None (*n* = 13)**	**Weak (*n* = 4)**	**Moderate (*n* = 17)**	**Strong (*n* = *16*)**
ica operon	1 (7.7)	1 (25.0)	6 (35.3)	13 (81.3)
atlE	13 (100.0)	4 (100.0)	17 (100.0)	16 (100.0)
embP	13 (100.0)	4 (100.0)	17 (100.0)	16 (100.0)
sesC	13 (100.0)	4 (100.0)	17 (100.0)	16 (100.0)
aap	1 (7.7)	4 (100.0)	16 (94.1)	15 (93.8)
arcA	1 (7.7)	1 (25.0)	10 (58.8)	8 (50.0)
bhp	3 (23.1)	1 (25.0)	9 (52.9)	3 (18.8)

Note: Biofilm ability was subdivided into four biofilm categories depending on the crystal violet OD: non-producer (OD<0.120), weak producer (0.120<OD<0.240), moderate producer (0.240<OD<0.480) and strong biofilm producer (OD>0.480).

Table S5 Association between plasmids containing *blaZ* and *mecA*, clinical outcome and biofilm formation in *S. aureus* strains

Plasmid	No of isolates (%)		P value[a]	No of isolates (%)		P value[a]
	Unresolved (n = 23)	Resolved (n = 22)		Non/weak biofilm (n = 8)	Strong biofilm (n = 37)	
Rep5a + *blaZ*	14 (58.3)	10 (41.7)	0.3001	4 (16.7)	20 (83.3)	0.8349
Rep16 + *blaZ*	20 (64.5)	11 (35.5)	0.0074**	4 (12.9)	27 (87.1)	0.2031
Rep7a + *mecA*	3 (75)	1 (25)	0.3167	- (0)	4 (100)	
Rep7c + *mecA*	6 (75)	2 (25)	0.1360	1 (12.5)	7 (87.5)	0.6668

Note: **$P \leq 0.01$ statistically significant, Chi-square test.
Only *S. aureus* strains positive for the presence of *blaZ* or *mecA* in the corresponding plasmid Rep5a, Rep16, Rep7a or Rep7c were included in the analysis.
